# Remarks on Reviewing

**Published:** 1809-12

**Authors:** 


Medical and Physical Journal.

VOL. XXII.] DECEMBER, 1809. [NO. 130.

Printed for R. PHILLIPS, by W. Thorne, Red Lion Court, Fleet Street, London.

### REMARKS ON REVIEWING.

THE most painful part of all our labours is the Critique we conceive it our duty to offer on the medical productions of the day. Though this part of our Journal is always anonymous, yet we are not so disingenuous as to wish to protect ourselves by such an evasion. On many occasions, if we are obliged to differ, and more particularly if circumstances oblige us to mark that difference with any severity, we have expressed a readiness to receive any explanation from the author or his friends.

The object of our Journal is the improvement of medical knowledge. This cannot be attained without the most scrupulous impartiality; and we conceive may be further aided by a mode of expression, which may not entirely discourage a young author, even if we find it necessary to reprove him. Towards veterans, less delicacy may be required. After having frequently appeared before the public, an author must be considered incorrigible if he sins from carelessness or ignorance; and it becomes necessary to mark his errors so strongly, that if *he* cannot feel reproof, the reader may not be misled.

Impressed with these sentiments, we very early communicated our wish that the critiques of a rival Journal should savour less of that severity with which a periodical publication from the same metropolis was at that time too justly accused. Whether in consequence of our hint, we know not, but are glad to perceive some amendment in that quarter. We should have left unnoticed another publication, the whole object of which is critical, had we not received the following paper from a gentleman, who has favoured us with his name. For some time, however, we had determined to avoid interfering in a contest with which, as Editors of the Journal, we were no way connected. If we have altered our intention, we must beg our readers to recollect: First, the great importance of the disease disease to which the controversy relates. Next, the general admission of surgeons, that the topical application of *iron* to sores deemed cancerous, as suggested by Mr. Carmichael, has been found highly serviceable; and, lastly, may we be permitted to add, that the ground-work of the practice being derived from the opinions of one of our Editors, many difficulties occurred to us in every attempt at reviewing Mr. Carmichael's "Essay on the Effect of Iron on Cancers;" we thought the present, therefore, a good opportunity of bringing it before our readers, from a name which, though not concealed from, is entirely new to us.

The reader will perceive, on perusing the communication which follows our remarks, that it bears a reference to other critiques in the same number in which Mr. Carmichael is reviewed. This induced us to read the whole number, that we might not commit ourselves, and be under the necessity of making an excuse on the plea of ignorance. On the perusal, however, we thought we could not better serve the writers of that work, than by exhibiting our Hibernian correspondent in his true form. We leave to our readers to determine what apology may be made for the warmth of friendship, for the national failing of a love of humour, and for the crudities of one apparently not much accustomed to write.

We wish we could say the same for those men who profess to avoid "offending Dr. Lamb's feelings by irony or ridicule," yet at the close of their remarks, gently consign him to a mad-house, the government of which is to be committed to these merciful managers. Their sympathy for Dr. Reeve is of the same kind, "who, as they are informed is a young man of much industry and some talents."—A critique, comprising exactly half a sheet, concludes (besides other expressions of condolence which we shall not transcribe) with remarking that all the information contained in his book, is collected from "modern works which are generally read."—Why then this wanton cruelty of eight pages of teazing? Would it not have been kind in these compassionate writers, to remind their readers of the best account of Cretinism ever yet published, and deemed worthy a place in the Philosophical Transactions? If it were thought necessary to mark in general terms their disappointment at the "Essay on the Torpidity of Animals," from the same ingenious writer, might they not have satisfied themselves with a few hints, by which the author might have conducted some future experiments with credit to himself, and advantage to the world?

The other authors referred to by our Correspondent, as harshly treated in the same Number, are, Dr. Arnold and M. Richerand. Of the first of these, we find it difficult to say any thing, because we may perhaps be included in one part of the accusation. It is however remarkable, if the Critic's opinion be just, that our Reviewer should not have received an order to prepare a puff for Dr. Arnold's publication; yet on inquiry, he assures us, that he never heard of the pamphlet till he was referred to the review of it. Without therefore noticing even the *manners* with which Dr. Arnold is treated, we shall select one other passage from the review of Dr. Reeve. The *knowing part* of our Readers will see more in it than we shall take the trouble of pointing out; our principal motive in transcribing it is, in order that it may prove introductory to our apology for Mr. Carmichael.

"We are not willing (say these gentlemen) to admit the plea of haste to comply with a supposed impatience, which the public has never felt; and of interruption by professional employment, the assertion of which is impertinent, however true. We formerly stepped out of our way, to remonstrate with a distinguished writer on syphilis, for the coyness with which he withheld important information, because, as he thought, much yet remained to be learned; and we may now express our approbation of the candour and boldness with which another eminent writer * has repeatedly submitted to the public, ideas confessedly immature. These remarks, however, relate to subjects of practical importanee, on which an early decision was required, and on which a just one could be formed by multiplied experience alone. In such cases, individual reputation might suffer by a full exposure of conjectures requiring to be verified, and an open avowal of imperfect information; but the interests of the profession must necessarily be advanced by the extension of curiosity and consequent research."

Thus one distinguished writer has done right in withholding part of his knowledge, and another in telling more than he knew; and this too on a disease for which we have a standard work, "founded on multiplied experience." And from whom? Let us answer in the words of these same gentlemen. In their remarks on M. Richerand, the only remaining piece alluded to by our Correspondent, is the following passage.—"Mr. Hunter's experiments have proved, beyond the possibility of doubt, that the matter of secondary venereal sores is not contagious, and the

* Mr. Abernethy.

the most respectable modern writers on this disease admit the fact." If then, after such an author had told us all *he* knew of this disease, it is still admissable for one "distinguished writer on syphilis" to hint that he knows more than he chooses to tell; and for another, "repeatedly to submit to the public, ideas confessedly immature;" Is one, who informs us of all he knows, and of a remedy, since generally approved, against the most deplorable of human miseries, to be held up as an object of public ridicule?

Before we take our leave of this subject, we think it proper to caution the reader against suspecting that the foot note in the quotation concerning a "distinguished" and an "eminent writer" is our addition. Who is meant by the first, we are still ignorant; nor should "we go out of our way" a second time, to cast so foul a reproach on any writer, whether distinguished or eminent, as to accuse him of intimating that he knew something more of the venereal disease than he chose to communicate to the world. We should even think it sufficient *once* to have hinted, that an "eminent writer" had repeatedly submitted to the public his confessedly "immature ideas."

In these remarks, we have endeavoured as much as possible to avoid the error of which we are accusing others. If we have failed, our intentions are at least honest; and without further apology, we shall offer the communication from which our suggestions originated.

